# Takotsubo Cardiomyopathy and Stressed Heart Morphology: Molecular, Hemodynamic, and Imaging Intersections

**DOI:** 10.3390/jcm14217638

**Published:** 2025-10-28

**Authors:** Omar Atef Abdelhamid Mahmoud, Boran Cagatay, Nagehan Kucukler, Fatih Yalcin, Mario J. Garcia

**Affiliations:** 1 Division of Cardiothoracic Surgery, Department of Surgery, University of Minnesota, Minneapolis, MN 55455, USA; 2 Department of Medicine, University of California at San Francisco, Cardiology UCSF Health, San Francisco, CA 94143, USA; 3 Department of Cardiology, School of Medicine, Akdeniz University, 07070 Antalya, Turkey; 4 Department of Cardiology, Montefiore Medical Center, Albert Einstein College of Medicine, Bronx, NY 10461-1900, USA

**Keywords:** Takotsubo Cardiomyopathy, basal septal hypertrophy, stressed heart morphology

## Abstract

Takotsubo Cardiomyopathy (TTC), often referred to as stress-induced or “broken heart” syndrome, is characterized by transient left ventricular dysfunction predominantly involving apical hypokinesia and basal hyperkinesia in the absence of obstructive coronary artery disease. Traditionally viewed as an acute and reversible phenomenon, accumulating evidence suggests that TTC may emerge from a preexisting myocardial substrate shaped by chronic stress and hemodynamic loading. Basal Septal Hypertrophy (BSH), a morphological finding commonly observed in elderly, hypertensive, or emotionally stressed individuals, has been increasingly recognized in patients with TTC. This hypertrophic pattern, often accompanied by dynamic contractile gradients and regional perfusion mismatch, reflects a broader adaptive remodeling process conceptualized as Stressed Heart Morphology (SHM). SHM encompasses the structural and functional myocardial responses to cumulative neurohormonal and mechanical stress, with BSH representing a key imaging marker within this spectrum. Advanced echocardiographic techniques, such as tissue Doppler imaging, speckle-tracking strain analysis, and stress echocardiography, consistently reveal overlapping features between SHM and TTC, including basal hyperkinesis, septal thickening, and inducible left ventricular outflow tract obstruction. These findings support a continuum in which SHM serves as a predisposing substrate for TTC, representing a stress-provoked clinical expression within a unified myocardial stress–response framework.

## 1. Introduction

Takotsubo Cardiomyopathy (TTC), also known as stress cardiomyopathy or “broken heart syndrome”, is characterized by transient left ventricular (LV) dysfunction that typically follows acute emotional or physical stress. It commonly presents with apical ballooning of the left ventricle, mimicking an acute myocardial infarction but without evidence of obstructive coronary artery disease. TTC is more prevalent in postmenopausal women and is often triggered by intense emotional events [1,2]. In addition to the previously established reasons outlined in the literature, it is also known that major diseases such as cancer can be triggered by pathways involving oxidative stress [3].

Epidemiological studies of emotional stress, anxiety, and major depression have similarly identified gender-related disparities. In particular, older women exhibit a higher prevalence of major depression and chronic anxiety compared to men [4].

Although not universally observed, many patients with TTC have a history of chronic hypertension. Interestingly, in approximately 30% of TTC cases, despite being classified as stress-induced cardiomyopathy, no identifiable acute emotional or physical stressor is reported [5].

In light of previous studies, we propose that TTC may not be merely an acute event, but rather the result of a chronic remodeling process under persistent pressure overload [6].

Chronic pressure overload can lead to myocardial remodeling, particularly basal septal hypertrophy (BSH), which may be related to the heart’s periodic sensitivity to sympathetic stimulation and is associated with quantitatively increased dynamics under stress [7]. The combination of BSH and a stress-induced catecholamine surge can exacerbate tissue energy consumption due to repetitive oxygen usage [8,9].

“Stressed Heart Morphology” (SHM) refers to structural myocardial adaptations that occur in response to chronic or acute stress, most commonly presenting as BSH or asymmetric septal thickening. SHM is frequently observed in elderly individuals, especially those with hypertension, increased afterload, or prolonged emotional stress. Both SHM and TTC are associated with regional myocardial contractile imbalances, typically basal hyperkinesis, which may lead to dynamic obstruction [10]. However, these geometric and functional aspects in hypertension represent the adaptive phase of hypertensive heart disease [6,10].

On the other hand, there is a wide range of reported myocardial tissue function under stress, ranging from hyperdynamism to transient ischemic dilation and TTC [10].

## 2. Methods

This review followed a narrative and integrative framework, combining evidence from clinical, experimental, imaging, and molecular research on Takotsubo Cardiomyopathy (TTC) and Stressed Heart Morphology (SHM). A comprehensive literature search was conducted across PubMed, Scopus, and Web of Science databases from inception through September 2025 using combinations of the following terms: “Takotsubo cardiomyopathy”, “stress-induced cardiomyopathy”, “basal septal hypertrophy”, “hypertensive remodeling”, “stressed heart morphology”, and “neurohormonal stress”.

Eligible studies included peer-reviewed clinical trials, observational studies, imaging analyses, animal models, and mechanistic investigations that addressed pathophysiology, hemodynamics, or imaging correlates of TTC and SHM. Reference lists of major articles were reviewed to identify additional relevant studies. No date restrictions were applied, and only English-language publications were included.

The review strategy aimed to integrate data from four major domains: (1) structural and functional imaging of TTC and SHM; (2) hemodynamic and perfusion alterations under acute or chronic stress; (3) molecular mechanisms linking catecholamine signaling, estrogen modulation, and myocardial remodeling; and (4) the interplay between emotional, mechanical, and neurohormonal stressors in shaping myocardial geometry. Evidence was synthesized qualitatively to construct an integrative model of stress-mediated cardiac remodeling.

## 3. Results

### 3.1. Overview of Literature Findings

A total of 78 relevant studies were identified, with 53 studies later on, encompassing human clinical data, experimental animal studies, and mechanistic research. Across these studies, TTC and SHM were shown to share overlapping structural, functional, and molecular characteristics. Imaging findings consistently revealed basal septal hypertrophy (BSH) and regional contractile heterogeneity as recurring features in hypertensive and stress-related cardiac remodeling.

### 3.2. Imaging and Hemodynamic Observations

Advanced echocardiographic and cardiac magnetic resonance imaging studies demonstrated that TTC and SHM display a continuum of myocardial remodeling. Both exhibit basal hyperkinesis and apical hypokinesis during stress, producing dynamic left ventricular outflow tract (LVOT) gradients and transient systolic dysfunction. These geometric patterns are accompanied by early reductions in global longitudinal strain and impaired diastolic relaxation, even before overt hypertrophy is apparent. Perfusion imaging revealed segmental flow mismatch between basal and apical regions, indicative of microvascular dysfunction in hypertrophied segments.

### 3.3. Molecular and Neurohormonal Mechanisms

Experimental studies highlighted catecholamine excess as a primary trigger for transient myocardial dysfunction. Excessive activation of β_2_-adrenergic receptors shifts intracellular signaling toward the Gαi pathway, leading to apical contractile depression as a cardioprotective adaptation. Activation of the PI3K–AKT survival pathway and estrogen’s modulatory role on adrenergic sensitivity were consistently demonstrated. Chronic stress models revealed persistent activation of the hypothalamic–pituitary–adrenal (HPA) axis, the renin–angiotensin–aldosterone system (RAAS), and inflammatory cascades involving interleukin-6 and tumor necrosis factor-alpha, all contributing to maladaptive remodeling and fibrosis.

### 3.4. Integration of Acute and Chronic Stress Effects

The cumulative evidence supports a unified continuum model linking SHM and TTC. Chronic hemodynamic stress promotes adaptive basal septal thickening and hypercontractility, while acute catecholamine surges may precipitate apical ballooning and reversible systolic dysfunction. Repeated exposure to subclinical stressors fosters microvascular rarefaction, metabolic imbalance, and myocardial fibrosis. In this context, SHM represents a compensated morphological adaptation phase, whereas TTC embodies its acute decompensated clinical manifestation triggered by transient neurohormonal overload.

Together, these findings emphasize the intricate interplay between mechanical load, neurohormonal activation, and emotional stress in shaping cardiac geometry and function within the TTC–SHM spectrum.

## 4. Discussion

### 4.1. Repeated Acute Stress, Takotsubo Cardiomyopathy, and Stressed Heart Morphology

Repeated acute stress induced significant increases in left ventricular wall thickness and chamber diameter in female rats, leading to a higher incidence of concentric left ventricular hypertrophy compared to male rats [10]. This finding may provide insight into the higher prevalence of TTC in women. Elevated corticosterone levels in female rats were linked to increased left ventricular wall thickness, while increased visceral fat was associated with hypertrophy in male rats [11].

Positron emission tomography (PET) studies in rodents demonstrated an approximately 47% increase in myocardial glucose uptake after repeated pharmacological stress [1]. However, this alteration in glucose consumption appears to drive significant changes in cardiac hemodynamics and structural remodeling. Repeated stress exposure has been shown to induce significant macroscopic alterations in cardiac structure. Notably, these structural changes exhibit sex-specific patterns: in female rats, both heart weight and blood volume decrease following repeated stress, whereas in males, stress leads to a marked increase in septal wall thickness accompanied by a reduction in ventricular cavity size [12]. Although repeated stress exposure leads to significant alterations in cardiac hemodynamic parameters (e.g., heart rate, blood pressure, ejection fraction) in rodents, evidence suggests that persistent subclinical dysfunction may continue even after the recovery period [1]. On the other hand, the long-term remodeling process is marked by persistent structural changes, including fibrosis and vascular alterations. Ongoing activation of remodeling pathways suggests a sustained maladaptive response [1].

### 4.2. Perfusion Abnormalities and Segmental Remodeling Under Cumulative Chronic Stress

Chronic arterial hypertension imposes a sustained hemodynamic burden on the left ventricle, initiating a pathophysiological cascade that originates at the microvascular level and ultimately culminates in global myocardial remodeling [13]. Microvascular rarefaction, characterized by diminished arteriolar density and compliance, attenuates myocardial perfusion reserve, such that pharmacological or exercise-induced stress frequently unmasks regional perfusion defects in the absence of obstructive epicardial coronary disease [14].

**Figure 1:**
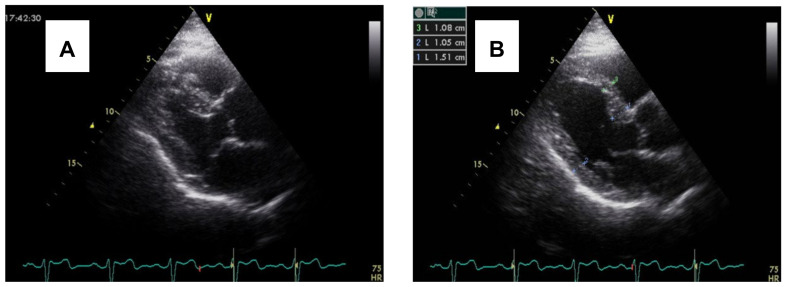
(**A**) Echocardiography shows a predominant regional LV septal base during end-diastole in a hypertensive patient with basal septal hypertrophy. (**B**) Protrusion of septal basal tissue into the LV outflow tract during end-systole in the same patient. (**B**) shows basal septal hypertrophy in a hypertensive patient.

Gated single-photon emission computed tomography and radionuclide imaging studies have demonstrated that hypertensive patients may exhibit a paradoxical juxtaposition of stress-induced hypoperfusion within hypertrophied segments and globally hyperdynamic systolic function. This highlights a perfusion demand mismatch that predisposes to microvascular angina, exertional dyspnea, or silent ischemia [15]. Progressive interstitial fibrosis and arteriolar wall thickening exacerbate this maladaptation, and concomitant elevations in circulating fibrosis biomarkers correlate with heightened risks of myocardial infarction, heart failure, and all-cause mortality [6] (Figure 1).

**Figure 2:**
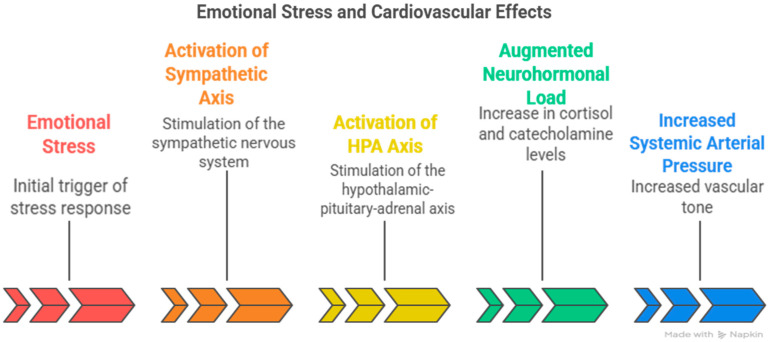
Shows how Superimposed emotional stress amplifies the deleterious cardiovascular effects of chronic hypertension by persistently activating the sympathetic and hypothalamic–pituitary–adrenal (HPA) axes, thereby augmenting systemic arterial pressure and neurohormonal load.

Emotional stress is widely recognized as one of the most common contributors to cardiac perfusion abnormalities and structural remodeling [16]. Superimposed emotional stress amplifies the deleterious cardiovascular effects of chronic hypertension by persistently activating the sympathetic and hypothalamic–pituitary–adrenal (HPA) axes, thereby augmenting systemic arterial pressure and neurohormonal load [17] (Figure 2).

To encapsulate the early geometric and functional consequences of these convergent stressors, we introduce the construct of SHM. SHM denotes the adaptive phase during which heterogeneous stress stimuli, mechanical, neurohumoral, and metabolic, produce a reproducible morphological signature: selective hypertrophy of the basal interventricular septum [18,19]. This phenotype is coupled to functional reprogramming that enables transient hypercontractility through mobilization of stored metabolic energy [1].

Chronic persistence or accumulation of stressors, however, precipitates a transition from adaptive hyperkinesis to maladaptive dysfunction, manifesting clinically as stress-provoked ischemia without angiographic coronary stenosis or as Takotsubo-like apical hypokinesia driven by segmental pressure gradients [20].

Importantly, the remodeling trajectory initiated by SHM remains regional long before global left ventricular hypertrophy becomes apparent, affording a critical therapeutic window for early intervention. Contemporary echocardiographic modalities, such as tissue Doppler imaging, speckle-tracking strain analysis, and exercise or dobutamine stress echocardiography, facilitate the detection of basal septal thickening [9], exaggerated regional contractile reserve, incipient dynamic left ventricular outflow tract (LVOT) gradients, subtle impairments in global longitudinal strain, and early diastolic dysfunction while overall ventricular mass remains normal [21].

Even individuals with borderline hypertension can demonstrate stress-induced septal hyperkinesis with near-cavity obliteration, a pattern that may be misinterpreted as a physiological athlete’s heart or as a false-positive ischemic response [21]. Accurate recognition of these signatures is essential, as rigorous blood pressure control and timely neurohumoral modulation can reverse concentric remodeling, regress interstitial fibrosis, and normalize mid-wall fiber shortening, thereby forestalling progression to overt heart failure [22].

In summary, chronic hypertension, potentiated by recurrent emotional stress, initiates a sequential remodeling process that is initially focal and compensatory yet ultimately global and fibrotic. SHM and its associated early imaging biomarkers, basal septal hypertrophy, stress-induced hypercontractility, microvascular perfusion heterogeneity, as well as strain and diastolic abnormalities, constitute an integrated framework for identifying patients who remain within the reversible phase of hypertensive heart disease, enabling preemptive therapeutic strategies aimed at mitigating long-term cardiovascular morbidity and mortality.

### 4.3. Acute Versus Chronic Stress Effect on the Heart and Blood Hemodynamics

Acute and chronic stress exert profoundly different effects on cardiovascular function and hemodynamics. Acute stress primarily triggers immediate adaptive responses, whereas chronic stress leads to pathological remodeling and disease progression. Understanding these distinct mechanisms is crucial for recognizing how psychological stress contributes to cardiovascular morbidity and mortality.

The major effect of acute stress involves rapid activation of the sympathetic–adrenal–medullary (SAM) axis within seconds to minutes, constituting the “alarm reaction” phase of the general adaptation syndrome [23]. This activation produces characteristic hemodynamic changes that prepare the body for immediate action [24].

Acute stress causes increases in heart rate and blood pressure through catecholamine release. Studies have shown that typical acute mental stress raises blood pressure by approximately 20–25 mmHg [25].

The hemodynamic timeline from acute to chronic stress begins with sympathetic neural activation. This activation forces the heart to increase its rate and contractility [26]. Studies demonstrated that prolonged stress increases the mean arterial blood pressure to cardiac output ratio [27]. This suggests that the impact of stress on vascular resistance intensifies over time, likely because the cardiac tissue fails to adapt, thereby initiating a remodeling process that culminates in ventricular dysfunction.

Both acute and chronic stress exert a significant influence on cardiac structure and function. Repeated acute stress, even when mild, has been shown to induce morphometric changes such as increased septal wall thickness and reduced left ventricular cavity width, particularly in males, indicating early features of concentric remodeling. Chronic stress is also associated with alterations in blood volume and hemoconcentration, which contribute to increased cardiovascular workload and disease risk. These findings underscore stress as a potent modifier of cardiac morphology, with sex-specific vulnerabilities that may help explain gender disparities in cardiovascular outcomes [12].

**Figure 3:**
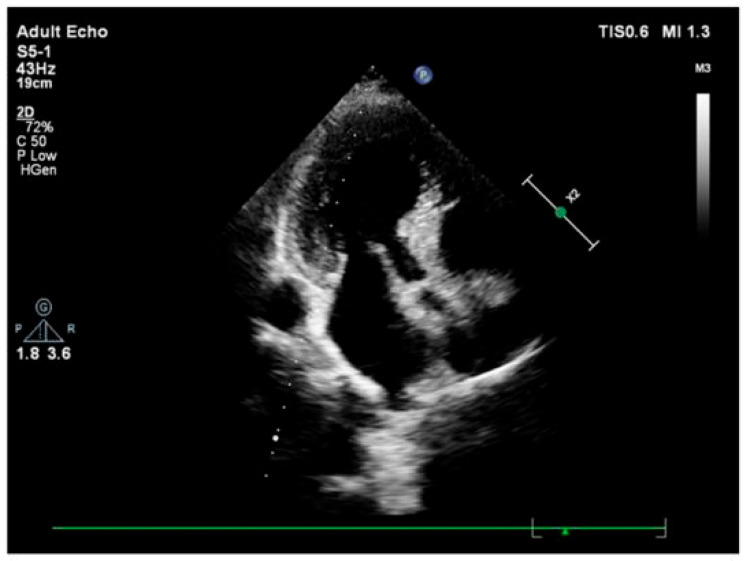
Shows a sharp curvature of more remarkable basal septal hypertrophy from apical four-chamber view during end-diastole in another hypertensive patient.

Repeated exposure to mild acute stress significantly reduces total blood volume, with a more pronounced and statistically robust effect observed in females. Notably, even after adjusting for body weight, stress accounted for 25% of the variance in blood volume among female rats, compared to 10% in males. These results indicate a sex-specific vulnerability to stress-induced hemoconcentration, which may increase cardiac workload and the risk of cardiovascular complications. Such hemodynamic alterations may underlie the pathophysiological link between chronic psychological stress and conditions such as hypertension, stroke, or coronary artery disease in humans [28] (Figure 3).

Acute stress may offer short-term myocardial protection, whereas chronic stress significantly increases vulnerability to ischemic injury. In rodent models, acute psychological stressors, such as forced swim or cold-restraint, have been shown to decrease myocardial infarct size and enhance contractile recovery after ischemia–reperfusion injury. These effects are attributed to ischemic preconditioning mediated by acute norepinephrine release and α_1_-adrenergic activation [29,30]. Conversely, chronic stress paradigms, including repeated restraint or social instability, have been found to increase infarct size and impair post-ischemic function. This is thought to involve sustained β-adrenergic stimulation, which exacerbates myocardial damage and hampers recovery [31].

Therapeutically, targeting stress-modulating systems such as endogenous opioid and gamma-aminobutyric acid (GABA)-ergic pathways may help mitigate stress-induced cardiac injury. In human populations, a large case–control study (INTERHEART) conducted across 52 countries, involving over 11,000 first myocardial infarction cases and 13,000 controls, reported that individuals experiencing permanent workplace stress had more than twice the risk of MI. Additionally, experiencing two or more acute stressful life events in the prior year was linked to a 48% increased MI risk [32].

**Figure 4:**
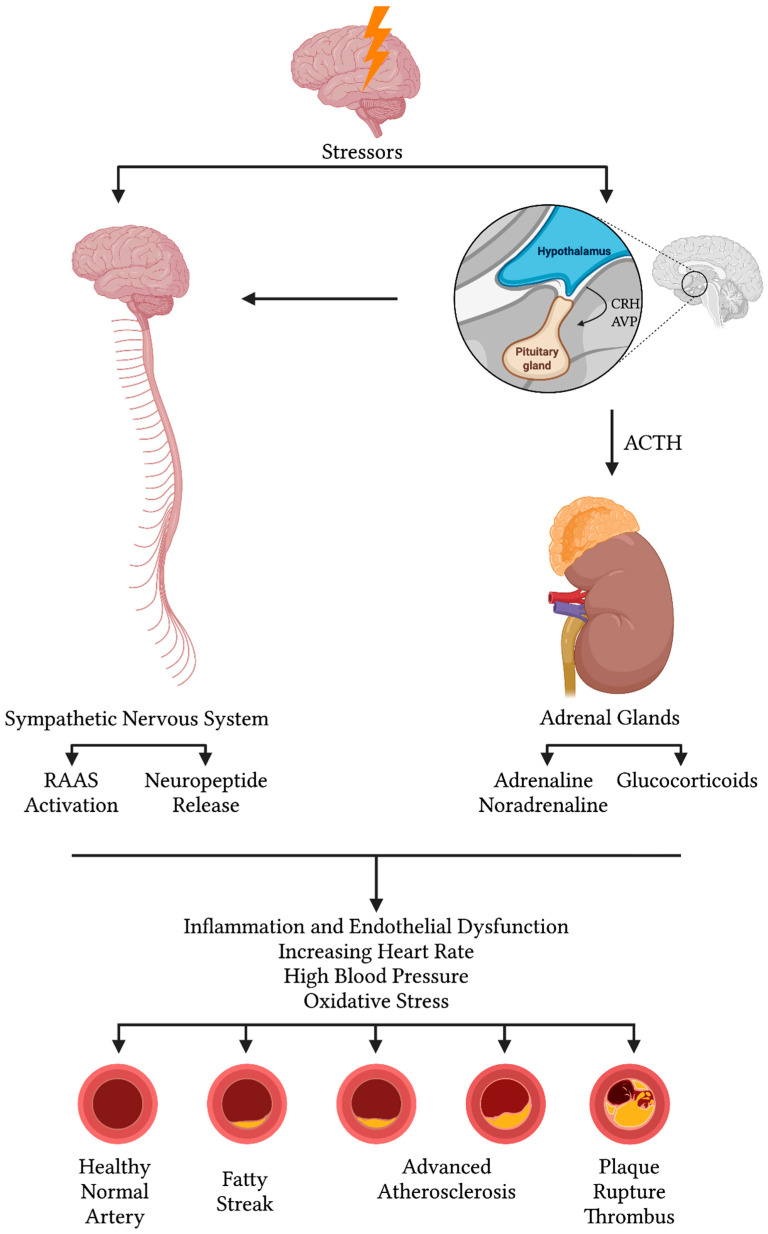
Shows the stress accumulating effect on HR, BP, and Inflammatory response through HPA, RAAS, Adrenaline, Noradrenaline, and Glucocorticoids release.

Mechanistically, acute stress activates both the HPA axis and the SAM system, releasing cortisol and catecholamines that mobilize energy, elevate heart rate and blood pressure, and temporarily enhance immune cell trafficking. However, chronic stress leads to sustained neuroendocrine activation, resulting in prolonged elevations of glucocorticoids and sympathetic tone. These effects drive endothelial dysfunction, insulin resistance, dyslipidemia, activation of the renin–angiotensin–aldosterone system (RAAS), and a shift toward enhanced systemic inflammation, marked by elevated levels of C-reactive protein, interleukin-6, and tumor necrosis factor-alpha. These changes promote oxidative stress, impair endothelial nitric oxide synthase activity, and accelerate atherosclerosis. Chronic stress may also impair the cholinergic anti-inflammatory reflex and activate the endocannabinoid system, further exacerbating metabolic and immune dysregulation [32] (Figure 4).

Together, these findings illustrate that while acute stress may induce a temporary cardioprotective preconditioning effect, chronic stress creates a persistent pro-atherogenic and pro-ischemic environment that substantially increases cardiovascular risk.

### 4.4. Molecular Mechanisms in Takotsubo Cardiomyopathy

TTC, also referred to as stress-induced cardiomyopathy, is characterized by transient left ventricular dysfunction, most notably apical ballooning. The underlying molecular mechanisms involve several intricate pathways. A central feature is adrenergic receptor signaling and biased agonism, wherein excessive catecholamine release during acute stress causes a shift in β-adrenergic receptor signaling from the Gαs to the Gαi pathway.

**Figure 5:**
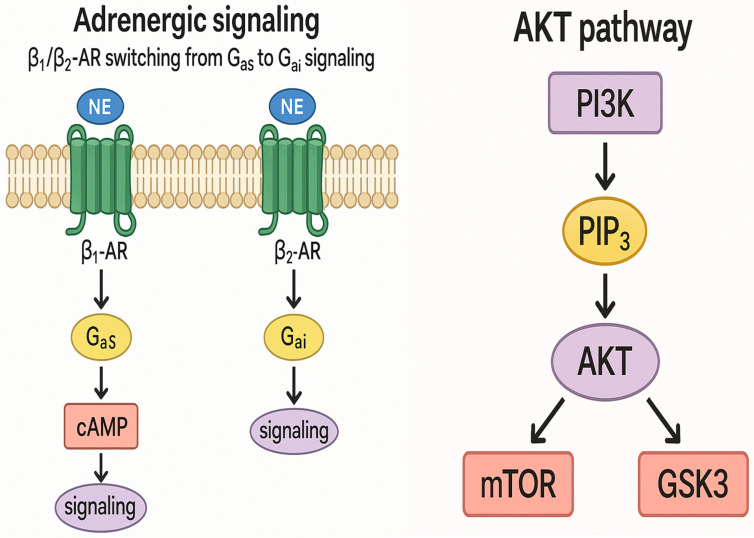
TTC apical ballooning underlying the mechanical mechanism involves Adrenergic signaling and the AKT pathway.

Under normal physiological conditions, β_1_- and β_2_-adrenergic receptors stimulate the Gαs pathway to enhance cyclic adenosine monophosphate (cAMP) production and myocardial contractility. However, in TTC, elevated catecholamine levels induce biased agonism, redirecting β_2_-adrenergic receptor signaling toward the Gαi pathway. This shift suppresses myocardial contractility (negative inotropy) and contributes to apical hypokinesia, serving as a cardioprotective adaptation against overstimulation and myocardial injury [33] (Figure 5).

Another key mechanism is activation of the phosphoinositide 3-kinase/protein kinase B survival pathway during the acute phase. This pathway supports cardiomyocyte survival by inhibiting apoptosis and enhancing metabolic activity. Downstream targets such as the mechanistic target of rapamycin and glycogen synthase kinase 3 regulate cellular proliferation and energy homeostasis, contributing to the reversible myocardial dysfunction observed in TTC [34] (Figure 5).

Estrogen plays a pivotal cardioprotective role in TTC pathogenesis. It modulates β-adrenergic receptor sensitivity, reduces calcium overload, and inhibits nuclear factor kappa-light-chain-enhancer of activated B cells-mediated inflammatory signaling. These protective effects explain the lower incidence of TTC in premenopausal women and highlight estrogen’s role in preserving myocardial integrity [35].

Microvascular reactivity and coronary vasospasm also contribute to TTC, despite the absence of obstructive coronary artery disease. Impaired endothelial function, heightened sympathetic tone, and the influence of vasoactive agents such as endothelin and reactive oxygen species disrupt coronary microcirculation, leading to localized ischemia and dysfunction. This mechanism is particularly evident in postmenopausal individuals, those with depressive disorders, or those using selective serotonin reuptake inhibitors [36].

**Figure 6:**
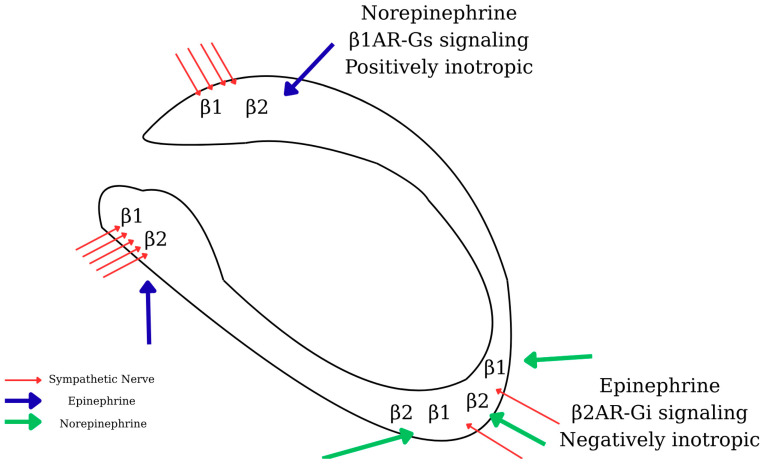
Representation of the regional differences in response to high catecholamine levels, explaining stress cardiomyopathy.

Regional perfusion abnormalities, especially in the apical myocardium, also play a critical role. Research has shown that the apex receives less perfusion than basal segments due to differences in gene expression, specifically, upregulation of structural genes and downregulation of metabolic genes. This results in segmental mismatch and impaired contractility in the apex [37] (Figure 6).

Furthermore, inflammation is increasingly recognized as a key player in TTC. Imaging studies using ultra-small superparamagnetic iron oxide particles have demonstrated macrophage accumulation in the myocardium during the acute phase. Elevated levels of pro-inflammatory cytokines such as interleukin-6 and chemokine ligand 1 suggest a systemic inflammatory response, which may contribute to the progression from transient myocardial dysfunction to chronic remodeling or even heart failure in some patients [38].

### 4.5. Stressed Heart Morphology and Takotsubo Cardiomyopathy Pathophysiological Correlation

A sudden increase in systemic blood pressure elevates left ventricular afterload and wall stress, contributing to the characteristic apical ballooning observed in TTC [39]. Notably, approximately 54% of patients with TTC also have a history of chronic hypertension [40]. Additionally, hypertensive patients tend to experience more frequent recurrences of Takotsubo episodes and demonstrate worse prognoses during these events [41].

Hemodynamic profiles in the acute phase of TTC are often unstable: some patients present with hypertensive crises, while others develop hypotension or cardiogenic shock due to impaired left ventricular systolic function [42].

In individuals with hypertension and basal septal hypertrophy, a catecholamine surge can provoke hyperdynamic basal contraction and dynamic obstruction of the LVOT. This further destabilizes blood pressure and increases myocardial oxygen demand. Simultaneously, the catecholamine surge activates the sympathetic nervous system and the RAAS, causing transient hypertension, systemic vasoconstriction, and elevated myocardial strain, which are key elements in the pathophysiology of TTC [8,9].

**Figure 7:**
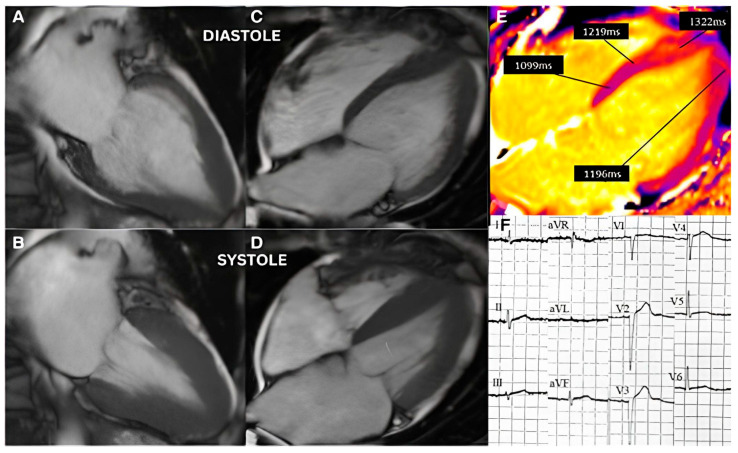
Shows a non-stress cMRI of a Caucasian woman with TTC, who then developed apical myocardial edema (pseudohypertrophy) mimicking apical HCM in the recovery phase. Baseline cardiac MRI. Diastolic (**A**,**C**) and systolic phases (**B**,**D**). Lack of basal to apical wall tapering (**C**). Elevated mid to apical T1 mapping times (The key feature that proves that this is a TTC rather than HCM) (**E**). Admission ECG with poor R-wave progression (**F**). Image permission taken from the author of reference [43].

SHM, by contrast, shows BSH patterns (one of SHM’s characteristics) arising in chronic stress states. Emerging research suggests that TTC and SHM share structural, functional, and mechanistic overlaps [43]. Both conditions display overlapping cardiac imaging characteristics, including basal hyperkinesis and apical hypokinesis, wall motion abnormalities visible on echocardiography or cardiac magnetic resonance imaging [43] (Figure 7).

SHM is associated with myocardial structural changes, including BSH and asymmetric septal thickening. It is commonly observed in elderly individuals, particularly those with hypertension or chronic emotional stress. Although its exact pathophysiology is not fully defined, current evidence suggests that cumulative stressors initiate characteristic geometric and functional remodeling in the myocardium [44,45].

The mechanisms underlying SHM are multifactorial, including chronic or exercise-induced hypertension [46], stress-induced alterations in brain cellular activity [47], persistent neurohormonal activation of cardiac sympathetic nerves [48], long-term variability in blood pressure [49], cognitive disorders [50], and adrenergic hyperactivity.

Experimental findings from mouse models under chronic stress conditions reveal an initial hyperactive stage in the basal septum that precedes left ventricular dysfunction. During this phase, a significant increase in intracavitary pressure gradients is observed [51]. Imaging studies in patients with TTC frequently reveal underlying basal septal thickening [52] or a sigmoid-shaped septum [53].

These findings support the hypothesis that SHM may constitute a predisposing phenotype for stress-induced cardiomyopathy. Recognizing this link is crucial, as SHM may increase the risk of progression to heart failure. This underscores the importance of close monitoring and targeted management of stress, hypertension, and myocardial workload in susceptible patients [8,9].

## 5. Conclusions

The remodeling trajectory of SHM begins in regional areas long before global left ventricular hypertrophy becomes apparent, offering a crucial therapeutic window [54]. Advanced echocardiographic techniques, including tissue Doppler imaging, speckle-tracking strain, and stress echocardiography (exercise or dobutamine-based), can identify basal septal thickening, heightened contractile reserve, early left ventricular outflow tract gradients, minor impairments in global longitudinal strain, and early diastolic dysfunction, even when overall ventricular mass appears normal [7,10].

Patients with borderline hypertension may exhibit stress-induced septal hyperkinesis with near-cavity obliteration, which can mimic athletic heart adaptations or produce false-positive ischemic responses [46,49]. Accurate interpretation of these patterns is essential, as timely blood pressure control and neurohumoral modulation can reverse concentric remodeling, reduce fibrosis, and restore myocardial fiber shortening, which prevents progression to overt heart failure.

Moreover, targeting stress-modulating systems, such as opioid and GABAergic pathways, may emerge as a future prophylactic strategy for reducing stress-induced cardiac injury [32].

## Data Availability

No new data were created or analyzed in this study. Data sharing is not applicable to this article.
